# Attitudes toward patients’ safety among healthcare professionals in the United Arab Emirates: A cross-sectional study

**DOI:** 10.1097/MD.0000000000047170

**Published:** 2026-01-16

**Authors:** Moien A.B. Khan, Sohrab Amiri, Iffat Elbarazi, Mohammed Salaheldin Ali Elsayed, Reem Al Falasi, Debasish Kar

**Affiliations:** a Department of Family Medicine, Health and Wellness Research Group, College of Medicine and Health Sciences, United Arab Emirates University, AL-Ain, United Arab Emirates; b Spiritual Health Research Center, Lifestyle Institute, Baqiyatallah University of Medical Sciences, Tehran, Iran; c Institute of Public Health, College of Medicine and Health Sciences, United Arab Emirates University, AL-Ain, United Arab Emirates; d Ambulatory Healthcare Services, Abu Dhabi, United Arab Emirates; e Community and Primary Care Research Group, University of Plymouth, UK; f Clinical Informatics and Health Outcomes Research Group, University of Oxford, UK.

**Keywords:** attitudes, healthcare professionals, patient safety, United Arab Emirates

## Abstract

This research aims to investigate healthcare professionals’ (HCPs) attitudes towards patient safety and to explore the role of various work-related factors that could be the barriers to safety-events reporting to modify these attitudes. A cross-sectional survey was conducted, involving 629 HCPs who are working across health care sectors in the United Arab Emirates. HCPs in the United Arab Emirates, comprising 71.5% women and 27.8% men, displayed a generally positive attitude towards patient safety (mean score = 3.91). Sub-comparisons indicated high scores for team functioning (4.58 ± 0.62) and low scores for professional incompetence as an error cause (2.86 ± 0.50). Common barriers to reporting safety events included uncertainty about procedures and fear of retribution. Confidence in reporting correlated with higher scores, while fear of reprisal yielded lower scores. Varied perceptions of medical errors’ implications were noted. Clear reporting procedures and event definitions impacted safety attitudes significantly. The study’s findings indicate an overall positive attitude towards patient safety among healthcare professionals. Promoting clear reporting protocols, addressing fear of reprisal, and providing ongoing education can improve patient safety attitudes. Longitudinal research should further explore these dynamics for sustained improvements in healthcare safety culture. These results emphasize the importance of integrating patient safety education into medical training programs.

## 1. Introduction

Patient safety is the prevention and reduction of risks, errors, and harm that occur to patients during the provision of health care. It is a fundamental aspect of high-quality care that protects patients and supports healthcare teams.^[1]^ The maintenance of high standards of patient safety is crucial for ensuring the reliability and effectiveness of healthcare systems globally.^[2]^ Since the influential report “To Err is Human” was published, patient safety has gained significant importance in worldwide healthcare conversations.^[1]^ Ensuring safety in healthcare provision by proactively identifying healthcare risks and mitigating them are a priority for the health sectors worldwide.

Medical errors are significant concerns for poor health outcomes. It is considered to be one of the primary contributors to mortality in the United States and worldwide.^[3]^ Economic burden and quality-of-life-years loss due to medical errors are substantial. Significant variation exists in error rates among different countries of the world including Europe and the Middle East, suggesting that there is a scope for lessons that could be shared to improve patient safety and to prevent avoidable adverse health outcomes.^[4,5]^ Most of the healthcare facilities in the developing world have not yet fully adopted essential patient safety measures, which is a global concern that requires comprehensive measures to protect patients.^[6,7]^ Lack of robust clinical governance guidance, professional accountability and cultural barriers are often attributed to poorer health outcomes in the Middle East, compared to Europe and the USA. However, there is scarcity of evidence to support or refute this assumption. Therefore, to address this knowledge gap and improve health outcomes, it is necessary to investigate healthcare professionals (HCPs) attitudes to patient safety and the barriers to modify these attitudes.

In contrast to developed countries, developing countries lag in implementing patients’ safety in clinical practice. To prevent avoidable harm and improve health outcomes, HCP’s views and attitudes to patients’ safety are crucial components.^[8]^ In developed countries, adherence to clinical governance guidelines and health and safety legislations are essential components of performance review and accountability. Therefore, the patients’ safety is embedded in professional practice and governed by organizational and national clinical governance framework, comprehensive risk assessment and mitigation protocol, health and safety legislation and mandatory reporting policies on “near-misses.” HCPs are also supported by annual appraisal to discuss their concerns and celebrate achievements. Organizational no-blame-culture and logistic support enhances team dynamics and facilitates engagement and adherence to safety and clinical governance protocols. Implementing robust regulations and establishing effective incident reporting systems is crucial for strengthening patient safety practices in hospitals. These tactics enhance healthcare practitioners’ consciousness of potential safety hazards and foster a culture where safety concerns and errors are freely acknowledged and dealt with.

Enhancing patient safety in hospitals is crucial for developing countries at the national level. An effective strategy should incorporate accreditation, certification, research, regulatory assistance, and ongoing education to establish a robust safety culture.^[9]^

The United Arab Emirates (UAE) provides a distinctive setting for studying patient safety. The healthcare system in the UAE, albeit resembling European models, possesses unique cultural and operational attributes that impact patient safety.^[10]^ This combination offers a valuable opportunity to investigate how the attitudes of healthcare personnel towards patient safety impact the safety culture inside healthcare institutions in the UAE.

The current study hypothesizes that HCPs in the UAE, when provided with conducive work conditions and robust incident reporting mechanisms, are inclined to exhibit favorable attitudes towards patient safety.

The main aim of this research is to examine the perspectives of healthcare professionals in the UAE about patient safety. The focus is on determining the factors that impact these perspectives and how they vary among different professional groups. This study seeks to gain insights into the dynamics of patient safety culture in the UAE by analyzing the correlation between supportive work environments, effective incident reporting procedures, and healthcare personnel’ attitudes towards patient safety.

This study aims to enhance comprehension regarding HCPs’ perception and involvement in patient safety culture by examining the influence of work-related factors and obstacles to incident reporting on their views.

## 2. Materials and methods

### 2.1. Study design and participants

This cross-sectional survey, adhering to Strengthening the Reporting of Observational Studies in Epidemiology criteria^[11]^ evaluated healthcare professionals’ attitudes and practices about patient safety and medical errors. A total of 629 actively practicing clinical professionals, including doctors, nurses, and physiotherapists, were recruited from various hospitals and healthcare centers around the UAE using convenience sampling. The recruitment was conducted by a team of trained researchers from various hospitals and healthcare centers within the UAE participation was voluntary, with each participant allocating 15 to 20 minutes to complete an anonymized survey. Confidentiality was emphasized, and participants had the right to withdraw at any point without consequences.

### 2.2. Sample size

A priori G × Power 3.1^[12,13]^ analysis for 1-way ANOVA comparing attitudes between groups of healthcare professionals with *f* = 0.20, α = 0.05, power = 0.80 gave N = 246. A more conservative *f* = 0.15 gave N = 348. For key 2-group comparisons among healthcare professionals (two-tailed *t* test, *d* = 0.30, α = 0.05, power = 0.80) the total N = 352. Allowing for nonresponse and subgroup imbalance, we targeted ≥420.

### 2.3. Inclusion and exclusion criteria

The study focused on health care professionals (doctors, nurses, and allied health care professionals). who were registered and actively practicing in the UAE. Excluded from the study were nonmedical healthcare professionals, administrative staff, medical students, HCPs licensed in the UAE but practicing elsewhere, or those not actively working and any incomplete survey responses.

Outcome measurement:

1. The study involved a 2-part questionnaire: Part 1 captured socio-demographic data, such as age, gender, and work-related information.
2. Part 2 used Attitudes to Patient Safety Questionnaire, which is a validated tool for capturing HCP’s attitudes to patients’ safety.^[14]^ The version used in this research included 25 items, presented as 5-point Likert scale (strongly agree to strongly disagree). Agree as well as strongly agree expressed positive responses to the item.^[15]^ Reverse scores were used with some items. Cronbach’s α coefficient of 0.75 has been reported for this questionnaire.^[15]^ The online questionnaire and a printed questionnaire circulated among health care professionals in the UAE.

### 2.4. Ethical considerations

This study was conducted in accordance with the ethical guidelines outlined in the Declaration of Helsinki, receiving approval from the Social Sciences Research Ethics Committee of the UAE University (Approval No. ERS_2020_7224). Participants were formally recruited via institutional email invitations distributed to healthcare professionals. Informed consent was obtained from all participants, who were assured of their anonymity and informed of the study’s objectives and procedures before participation. No financial or nonfinancial incentives were offered for participation in the study.

### 2.5. Statistical analysis

Descriptive statistics were used to summarize the demographic characteristics and employment status of participants. Analysis were conducted using SPSS 26.^[16]^ Inferential statistics, including *t* tests and ANOVA, were applied to explore differences in attitudes towards patient safety based on demographic variables and reported barriers to safety event reporting. The significance level was set at *P* < .05 for all statistical tests.

## 3. Results

After institutional email invitations, 629 surveys were included in the analysis, with a nonresponse rate of approximately 40% mainly attributed to workload and scheduling. Among the total 629 HCPs included in this study, 71.5% were women, and 27.8% were men and 0.07% did not report their gender. Most participants (93.8%) were between the age of 20 to 30 years. The information related to the employment status of the participants is listed in Table 1.

**Table 1:** Participants’ demographics characteristics and employment status.

Variable	Subgroup	N	%
	Total	629	100
Gender	Female	453	72
	Male	176	28
Age	>40	3	0.5
	20–25	331	52.6
	26–30	259	41.2
	31–35	30	4.8
	36–40	6	1.0
In your staff position, do you typically have direct interaction or contact with patients?	No	54	8.6
	Yes	575	91.4
Professional work experience (yr)	<1	339	53.9
	1–5	266	42.3
	6–20	24	3.8
Hospital experience (yr)	<1	186	29.6
	1–5	421	66.9
	≥6	22	3.5
Work in the same unit experience (yr)	<1	438	69.6
	1–5	178	28.3
	6–20	13	2.1
Working hours per week	<20	66	10.5
	20–39	178	28.3
	40–59	316	50.2
	60–79	51	8.1
	≥80 h	18	2.9
Have you had patient safety training in your workplace	No	237	37.7
	Yes	392	62.3
Would you be interested in receiving patient safety training	No	26	4.1
	Yes	603	95.9

Regarding barriers to reporting patient safety events within clinical practices, several key factors were identified (Table 2). Among these, uncertainty about the reporting process emerged as a notable concern, with 38.6% of respondents occasionally unsure of how to submit a report, and a combined 24.8% reporting this uncertainty often or most of the time. The time commitment required for submitting reports was another significant barrier, with 44.0% of healthcare professionals citing it as an occasional issue, and 27.0% experiencing it often or most of the time.

**Table 2:** Barriers to reporting patient safety events.

At your clinical practice, how often are the following barriers to reporting safety events	Most of the time N (%)	Often N (%)	Sometimes N (%)	Seldom N (%)
Unsure how to submit a report	53 (8.4%)	103 (16.4%)	243 (38.6%)	230 (36.6%)
Time it takes to submit an event report	41 (6.5%)	129 (20.5%)	277 (44.0%)	182 (28.9%)
Unsure of what is considered a patient safety event	39 (6.2%)	106 (16.9%)	230 (36.6%)	254 (40.4%)
Fear of retribution to self	42 (6.7%)	109 (17.3%)	239 (38.0%)	239 (38.0%)
Fear of retribution to others	44 (7.0%)	112 (17.8%)	269 (42.8%)	204 (32.4%)
Fear of violating hierarchy	58 (9.2%)	119 (18.9%)	258 (41.0%)	194 (30.8%)
Lack of perceived change due to submitting a patient safety event	37 (5.9%)	125 (19.9%)	264 (42.0%)	203 (32.3%)
Medical error seen as a sign of incompetence	54 (8.6%)	116 (18.4%)	216 (34.3%)	216 (34.3%)

The clarity regarding what constitutes a patient safety event also posed challenges, with 36.6% facing this uncertainty sometimes, and a total of 23.1% often or most of the time. Fears related to retribution both towards oneself (38.0% sometimes concerned) and others (42.8% sometimes concerned) along with apprehensions about violating hierarchical norms (41.0% sometimes concerned), further hindered reporting. These fears were also frequently or most of the time a concern for 24.0% (self) and 24.8% (others), and 28.1% (hierarchy) of respondents, respectively.

The perceived lack of change following the submission of a patient safety event deterred reporting for 42.0% of individuals sometimes, with 25.8% feeling this way often or most of the time. The view of medical errors as indicative of incompetence was another barrier, with 34.3% of healthcare professionals occasionally perceiving it this way, and 27.0% often or most of the time.

The mean attitudes to patient safety score across all participants was 3.91 (standard deviation [SD] = 0.32), reflecting a generally positive stance towards patient safety. Scores for men and women were nearly identical, at 3.92 (SD = 0.32) and 3.92 (SD = 0.31), respectively. Among specific dimensions, “team functioning” received the highest average score (4.58 ± 0.62), while “professional incompetence as an error cause” received the lowest (2.86 ± 0.50). The statement “teaching residents about patient safety should be an important priority in residency training” garnered the most positive responses (Supplemental Digital Content 1, Supplemental Digital Content, https://links.lww.com/MD/R127).

The Attitudes to Patient Safety Questionnaire (see Table 3) assessed various dimensions of healthcare professionals’ attitudes. High mean scores were seen in areas such as patient safety training (3.98), confidence in error reporting (4.19), recognition of working hours as a factor in errors (4.43), and team functioning (4.58). The importance of patient safety within medical curricula also scored positively (4.03). However, lower perceptions were noted for “professional incompetence as an error cause” (mean = 2.86), with some respondents attributing errors to carelessness by nurses (13.2%) or doctors (47.8%). Notably, only 30.7% agreed that doctors should disclose errors to patients only when harm has occurred, indicating varied beliefs around error disclosure.

**Table 3:** Means, standard deviation of attitudes to patient safety questionnaire*.

APSQ item	Overall	Men	Women
Overall questionnaire score	3.91 (0.32)	3.92 (0.32)	3.91 (0.32)
Patient safety training received	3.98 (0.88)	3.94 (0.86)	3.99 (0.88)
Error-reporting confidence	4.19 (0.82)	4.23 (0.75)	4.18 (0.85)
Working hours as an error cause	4.43 (0.67)	4.40 (0.67)	4.45 (0.67)
Error inevitability	4.14 (0.63)	4.15 (0.67)	4.14 (0.62)
Professional incompetence as an error cause	2.86 (0.50)	2.81 (0.53)	2.87 (0.48)
Disclosure responsibility	3.65 (0.57)	3.61 (0.53)	3.67 (0.58)
Team functioning	4.58 (0.62)	4.64 (0.57)	4.56 (0.63)
Patient involvement in reducing errors	3.98 (0.83)	4.14 (0.75)	3.92 (0.85)
Importance of patient safety in the curriculum	4.03 (0.57)	4.04 (0.59)	4.02 (0.56)

APSQ = Attitudes to Patient Safety Questionnaire.

* Higher scores indicated more affirmative responses.

Professional experience within healthcare settings showed varied impacts on attitudes toward patient safety (Table 4). While those with <1 year of professional experience had slightly different attitudes compared to those with 1 to 5 years and 6 to 20 years of experience, these differences were not statistically significant (*P* = .18 and *P* = .68, respectively). Although minimal, these trends suggest a potential influence of experience length on safety attitudes, warranting further investigation.

**Table 4:** Comparison attitudes to patient safety scores based on participants’ working conditions.

Working condition	N	Mean (SD)	Reference group	Mean difference	*P*-value	95% CI
Professional work experience (yr)	339 vs 266	3.90 (0.32) vs 3.94 (0.32)	<1 vs 1–5	−0.05	.18	−0.11 to 0.01
	339 vs 24	3.90 (0.32) vs 3.95 (0.36)	<1 vs 6–20	−0.06	.68	−0.22 to 0.10
Hospital experience (yr)	186 vs 421	3.85 (0.34) vs 3.95 (0.31)	<1 vs 1–5	−0.09	.003	−0.16 to −0.03
	186 vs 22	3.85 (0.34) vs 3.97 (0.34)	<1 vs 6–20	−0.12	.25	−0.29 to 0.05
Work in the same unit (yr)	438 vs 178	3.91 (0.32) vs 3.93 (0.33)	<1 vs 1–5	−0.02	.83	−0.08 to 0.05
	438 vs 13	3.91 (0.32) vs 3.90 (0.43)	<1 vs 6–20	0.02	.98	−0.20 to 0.23
Working hours per week	66 vs 18	3.90 (0.32) vs 4.00 (0.20)	<20 vs >80	−0.10	.75	−0.34 to 0.13
	178 vs 316	3.84 (0.33) vs 3.96 (0.32)	20–39 vs 40–59	−0.12	.001	−0.20 to −0.04

CI = confidence interval, SD = standard deviation.

Hospital experience had a notable impact on attitudes toward patient safety. A significant difference was observed between individuals with <1 year of hospital experience and those with 1 to 5 years (*P* = .003), with the latter group showing a mean increase of 0.09 in positive attitudes toward patient safety. This suggests that early hospital experience plays a critical role in shaping safety attitudes. However, when comparing those with <1 year of experience to those with 6 to 20 years, a negative mean difference of −0.12 was noted, though this was not statistically significant (*P* = .25). These findings indicate that the most pronounced shift in attitudes may occur during the early years of hospital work.

Working hours significantly influenced attitudes toward patient safety, though effects varied across groups. A notable difference was found between individuals working 20 to 39 hours and those working 40 to 59 h/wk, with the latter group showing lesser positive attitudes (mean difference = −0.12, *P* = .001), suggesting that extended hours may negatively impact patient safety perspectives. However, the comparison between individuals working fewer than 20 hours and those working over 80 hours showed a nonsignificant mean difference of −0.10 (*P* = .75), reflecting the complex nature of work hours on safety attitudes where extreme workloads did not yield straightforward effects.

Furthermore, hospital tenure showed some impact, with participants having 1 to 5 years of experience displaying slightly higher positive attitudes compared to those with <1 year. No significant differences in attitudes were observed based on professional experience or unit tenure. In addition, gender and age showed no significant effect on patient safety attitudes, indicating a broadly consistent perspective across these demographic variables (not reported in table).

As shown in Table 5, health care professionals who had the least barriers to reporting safety had more affirmative responses to attitudes to patient safety. The comparison of Attitudes to Patient Safety scores based on barriers to reporting safety events revealed several noteworthy findings. Participants who were often unsure of how to submit a report tended to exhibit slightly higher mean scores compared to those who were seldom unsure, suggesting a potential correlation between confidence in reporting procedures and positive attitudes towards patient safety. Conversely, individuals who often experienced a fear of retribution to themselves or others displayed lower mean scores than those who seldom experienced such fears, indicating a negative impact on attitudes towards safety when fear is prevalent. Similarly, participants who often felt uncertain about what constitutes a patient safety event showed lower mean scores compared to those who seldom experienced such uncertainty, reflecting a potential link between clarity of definitions and positive attitudes towards safety. However, no significant differences were observed in mean scores based on the time it takes to submit an event report or the fear of violating hierarchy. Likewise, increased scrutiny threatening medical autonomy and medical errors seen as a sign of incompetence did not significantly affect attitudes towards patient safety, suggesting that these factors may not strongly influence overall perceptions of safety.

**Table 5:** Comparison attitudes to patient safety scores based on barriers to reporting safety*.

Barrier	Comparison	Reference group	N	Mean (SD)	Mean difference	*P*-value	95% CI
Unsure how to submit a report	Often vs seldom	Often	103 vs 230	3.83 (0.38) vs 3.99 (0.30)	−0.15	<.001	−0.25 to 0.06
Time it takes to submit an event report	Often vs seldom	Often	129 vs 182	3.83 (0.34) vs 4.00 (0.31)	−0.17	<.001	−0.26 to 0.07
Unsure of what is considered a patient safety event	Often vs seldom	Often	106 vs 254	3.78 (0.36) vs 3.99 (0.31)	−0.21	<.001	−0.31 to 0.12
Fear of retribution to self	Often vs seldom	Often	109 vs 239	3.82 (0.35) vs 4.00 (0.29)	−0.18	<.001	−0.28 to 0.09
Fear of retribution to others	Often vs seldom	Often	112 vs 204	3.87 (0.35) vs 3.99 (0.30)	−0.12	.008	−0.22 to 0.02
Fear of violating hierarchy	Often vs seldom	Often	119 vs 194	3.87 (0.34) vs 3.97 (0.29)	−0.10	.037	−0.20 to 0.00
Medical error seen as a sign of incompetence	Often vs seldom	Often	116 vs 216	3.86 (0.34) vs 3.99 (0.30)	−0.13	.002	−0.23 to 0.04
Increased scrutiny threatens medical autonomy	Often vs seldom	Often	115 vs 227	3.83 (0.35) vs 3.99 (0.31)	−0.16	<.001	−0.25 to 0.07

CI = confidence interval, SD = standard deviation.

* Dependent variable attitudes to patient safety.

Professionals who often reported being unsure about how to submit a safety event or felt that the time required to submit an event report was burdensome exhibited lower attitudes towards patient safety, with mean differences of −0.15 (*P* < .001) and −0.17 (*P* < .001), respectively. This underscores the critical need for healthcare organizations to streamline reporting processes and provide clear guidance to staff.

Furthermore, fears associated with reporting, such as the fear of retribution towards oneself or others were also identified as significant barriers. Specifically, the fear of retribution to self-resulted in a mean difference in attitudes of −0.18 (*P* < .001), and the fear of retribution towards others led to a mean difference of −0.12 (*P* = .008). These findings highlight the importance of creating a nonpunitive reporting culture that supports open communication and reassures staff that reporting errors will not result in negative consequences.

Additionally, concerns about violating hierarchical norms within healthcare settings were shown to negatively impact attitudes, with a mean difference of −0.10 (*P* = .037), suggesting that addressing hierarchical barriers to communication and reporting is essential for fostering a safer patient environment. Misconceptions regarding medical errors, particularly the belief that errors are a sign of incompetence, significantly lowered safety attitudes, with a mean difference of −0.13 (*P* = .002). This indicates a need for education and culture change initiatives that reframe errors as opportunities for learning rather than as failures.

Lastly, the perception that increased scrutiny threatens medical autonomy was associated with lower safety attitudes, demonstrating a mean difference of −0.16 (*P* < .001). This reveals the necessity for healthcare organizations to manage perceptions of scrutiny positively, emphasizing its role in improving patient care rather than as a punitive measure.

## 4. Discussion

The current study provides significant insights into healthcare professionals’ attitudes towards patient safety within the UAE, highlighting the various factors that shape these perspectives. The study identified several key barriers to reporting patient safety events, including uncertainty about the reporting process, time commitment, clarity of what constitutes a patient safety event, and fears of retribution and hierarchical violations. Also, it found notable trends in attitudes based on professional work experience, hospital experience, work in the same unit, and working hours per week. Participants who were confident in the reporting process and experienced less fear of retribution had more positive attitudes towards patient safety. The study suggests that to improve patient safety culture, hospitals should ensure clear reporting processes, reduce barriers to reporting, and provide robust patient safety training. The results highlight the need for healthcare organizations to streamline reporting processes and provide clear guidance to staff, as professionals who were often unsure about how to submit a safety event or felt that the time required to submit an event report was burdensome exhibited lower attitudes towards patient safety. This finding is consistent with the previous research, which emphasizes the importance of efficient reporting mechanisms in fostering a positive safety culture.^[17]^

Fears associated with reporting, such as fear of retribution towards oneself or others, were identified as significant barriers, negatively impacting attitudes towards patient safety. Specifically, the fear of retribution resulted in a notable decrease in positive safety attitudes like previous study.^[18]^ This highlights the critical need for creating a nonpunitive reporting culture that supports open communication and reassures staff that reporting errors will not result in negative consequences.^[19]^

Furthermore, concerns about violating hierarchical norms within healthcare settings were shown to negatively impact attitudes, suggesting that addressing hierarchical barriers to communication and reporting is essential for developing a safer patient environment.^[20]^ Misconceptions regarding medical errors, particularly the belief that errors are a sign of incompetence, significantly lowered safety attitudes, indicating a need for education and culture change initiatives that reframe errors as opportunities for learning rather than as failures.^[21]^ Hence, introducing national legislation mandating confidential and nonpunitive incident reporting, along with user-friendly systems and regular feedback, would drive improvements and promote a culture of safety and continuous learning in healthcare settings.^[22]^

The study found a significant positive correlation between confidence in the reporting process and favorable attitudes towards patient safety. Participants who exhibited lower levels of fear of retribution displayed more positive attitudes, suggesting that to improve patient safety culture, hospitals should establish transparent reporting procedures, minimize obstacles to reporting, and offer comprehensive patient safety training.^[23]^ The high scores in team functioning and error-reporting confidence particularly highlight the essential role of collaborative team dynamics and open communication in cultivating a safety-centered culture.^[24]^

The findings also revealed significant patterns in attitudes based on professional experience, hospital experience, work in the same unit, and weekly working hours. These patterns suggest that continuous education and support for healthcare professionals throughout their careers can help maintain and enhance positive attitudes towards patient safety.^[15]^ Notably, individuals working fewer hours per week displayed lower mean scores related to patient safety attitudes, potentially due to less integration into the team or missing critical safety training and updates that full-time employees receive.^[25]^

To address these barriers and improve patient safety culture, healthcare institutions should focus on developing and implementing strategies that promote open communication, provide comprehensive training on reporting procedures, and implement nonpunitive approaches to error reporting. Overcoming communication breakdowns and addressing cultural variations in openness can help ensure that healthcare professionals feel empowered and safe to report incidents, thereby fostering a more transparent and learning-oriented environment.^[17]^

A significant strength of this study lies in its broad examination of healthcare professionals’ attitudes towards patient safety across various dimensions, providing a holistic view of the prevailing safety culture within the UAE. The utilization of a validated questionnaire enhances the reliability of the findings, offering a robust platform for understanding and improving patient safety practices. Moreover, the study’s focus on barriers to safety event reporting and the correlation between hospital experience and safety attitudes presents new insights into the factors influencing patient safety culture.

However, the study is not without limitations. The reliance on self-reported data may introduce bias, as participants might have provided socially desirable responses, especially concerning sensitive topics like error reporting and professional incompetence. In addition, the cross-sectional design limits the ability to establish causality between healthcare professionals’ attitudes and patient safety outcomes. Another limitation is the geographical concentration of the study within the UAE, which may restrict the generalizability of the findings to other healthcare contexts with different cultural and operational characteristics.

To address these limitations in future research, employing probabilistic sampling techniques could enhance the representativeness and generalizability of the results. Implementing longitudinal study designs would allow for the examination of causal relationships and the tracking of changes in attitudes towards patient safety over time. Furthermore, complementing self-reported data with objective measures, such as direct observations or institutional safety metrics, could provide a more comprehensive and accurate picture of the safety culture within healthcare settings. In summary, this study provides valuable insights into the attitudes of healthcare professionals towards patient safety in the UAE, revealing both the strengths and areas for improvement within the current safety culture. Addressing the identified limitations and leveraging the strengths can pave the way for enhanced patient safety practices. The implications for clinical practice, research, and education underscore the multidimensional approach required to foster a culture of safety that supports both healthcare professionals and patients.

Future research should investigate the effectiveness of specific interventions designed to improve safety culture, such as leadership training, team-building exercises, and error-reporting systems. Employing longitudinal study designs and complementing self-reported data with objective measures could provide a more comprehensive and accurate picture of the safety culture within healthcare settings.

## 5. Conclusion

The study’s findings highlight the crucial need for a supportive environment that promotes open communication and collaborative teamwork to improve patient safety. Healthcare institutions should focus on developing and implementing strategies to reduce barriers to reporting safety events, thereby facilitating a culture of transparency and continuous improvement. Integrating patient safety training into ongoing professional development programs can equip healthcare workers with the necessary competencies to navigate complex clinical environments safely. Furthermore, the study findings underscore the importance of tailored strategies to enhance patient safety culture across different groups within healthcare settings. For instance, targeted interventions might be necessary to ensure part-time or reduced-hour workers are fully integrated into patient safety initiatives. Additionally, continuous education and engagement strategies could help sustain and improve attitudes towards patient safety among all healthcare professionals, regardless of their tenure or working hours.

Future research should also investigate the effectiveness of specific interventions designed to improve safety culture, such as leadership training, team-building exercises, and error-reporting systems.

## Acknowledgments

We thank the participating healthcare professionals and site coordinators for their support in survey distribution and data collection.

## Author contributions

**Conceptualization:** Moien A.B. Khan, Sohrab Amiri, Iffat Elbarazi, Mohammed Salaheldin Ali Elsayed, Reem Al Falasi, Debasish Kar.

**Data curation:** Moien A.B. Khan, Sohrab Amiri.

**Formal analysis:** Moien A.B. Khan.

**Writing – original draft:** Sohrab Amiri, Iffat Elbarazi, Mohammed Salaheldin Ali Elsayed, Reem Al Falasi, Debasish Kar.

**Writing – review & editing:** Sohrab Amiri.

## Supplementary Material


