# Supplementary material for: Attitudes toward patients’ safety among healthcare professionals in the United Arab Emirates: A cross-sectional study
Source: Medicine (Baltimore). 2026 Jan 16;105(3):e47170. doi: 10.1097/MD.0000000000047170 (PMC12826262; doi:10.1097/MD.0000000000047170)
Supplement: Supplementary file 1 [file medi-105-e47170-s001.docx]

**Supplemental Digital Content 1:** Means, standard deviation and % of positive response of Attitudes to Patient Safety Questionnaire**

| Attitudes to Patient Safety Questionnaire | | | | | | | | | | |
| --- | --- | --- | --- | --- | --- | --- | --- | --- | --- | --- |
|  | | Overall | | | Men | | | Women | | |
|  | | Mean | SD | % of positive  response | Mean | SD | % of positive  response | Mean | SD | % of positive  response |
| Overall Questionnaire | | 3.91 | .32 |  | 3.92 | .32 |  | 3.92 | .31 |  |
| Patient safety training received | | 3.98 | .88 |  | 3.94 | .87 |  | 4.00 | .88 |  |
| 1 | My training has prepared me to understand the causes of medical errors | 3.96 | .975 | 69.3% | 3.97 | .940 | 68% | 3.96 | .983 | 68.9% |
| 2 | I have a good understanding of patient safety issues as a result of my undergraduate medical training. | 4.00 | .985 | 72.7% | 3.93 | .994 | 74.6% | 4.04 | .978 | 74.6% |
| Error-reporting confidence | | 4.19 | .82 |  | 4.23 | .75 |  | 4.19 | .84 |  |
| 3 | I would feel comfortable reporting any errors I had made no matter how serious the outcome had been for the patient | 4.24 | .992 | 80.6% | 4.26 | .928 | 77.7% | 4.24 | 1.009 | 81.8% |
| 4 | I would feel comfortable reporting any errors other people had made, no matter how serious the outcome had been for the patient | 3.94 | 1.04 | 70.1% | 4.02 | .950 | 72% | 3.92 | 1.073 | 69.3% |
| 5 | I am confident I could talk openly to my supervisor about an error I had made if it had resulted in potential or actual harm to my patient. | 4.42 | .875 | 86.8% | 4.41 | .885 | 85.1% | 4.42 | .860 | 87.5% |
| Working hours as an error cause | | 4.43 | .67 |  | 4.39 | .67 |  | 4.45 | .66 |  |
| 7 | Shorter shifts for doctors will reduce medical errors | 4.45 | .811 | 87.1% | 4.38 | .881 | 84% | 4.47 | .784 | 88.2% |
| 8 | By not taking regular breaks during shifts doctors are at an increased risk of making errors. | 4.45 | .790 | 87.1% | 4.41 | .803 | 84% | 4.48 | .770 | 88.5% |
| 9 | The number of hours doctors work increases the likelihood of making medical errors. | 4.41 | .832 | 85.5% | 4.41 | .789 | 84.6% | 4.42 | .836 | 86% |
| Error inevitability | | 4.14 | .63 |  | 4.14 | .67 |  | 4.14 | .62 |  |
| 10 | Even the most experienced and competent doctors make errors. | 4.52 | .719 | 91.3% | 4.52 | .726 | 90.3% | 4.53 | .700 | 91.8% |
| 11 | A true professional does not make mistakes or errors* | 3.96 | 1.19 | 71.1% | 3.98 | 1.19 | 72.5% | 3.94 | 1.20 | 70.2% |
| 12 | Human error is inevitable | 3.96 | 1.04 | 66.6% | 3.93 | .992 | 62.8% | 3.97 | 1.06 | 68% |
| Professional incompetence as an error cause | | 2.86 | .50 |  | 2.82 | .53 |  | 2.87 | .48 |  |
| 14 | Most medical errors result from careless nurses | 2.28 | 1.11 | 13.2% | 2.33 | 1.09 | 11.4% | 2.26 | 1.12 | 14% |
| 15 | If people paid more attention at work, medical errors would be avoided* | 2.27 | .98 | 10.8% | 2.2286 | 1.01 | 10.3% | 2.30 | .976 | 11.1% |
| 16 | Most medical errors result from careless doctors* | 3.39 | 1.07 | 47.8% | 3.3714 | 1.11122 | 46.3% | 3.4067 | 1.06 | 48.5% |
| 17 | Medical errors are a sign of incompetence* | 3.48 | 1.07 | 52.1% | 3.3486 | 1.14404 | 43.4% | 3.5378 | 1.05 | 55.8% |
| Disclosure responsibility | | 3.65 | .57 |  | 3.61 | .53 |  | 3.67 | .59 |  |
| 18 | It is not necessary to report errors which do not result in adverse outcomes for the patient* | 3.82 | 1.19 | 65.5% | 3.77 | 1.27 | 66.9% | 3.85 | 1.17 | 65.5% |
| 19 | Doctors have a responsibility to disclose errors to patients only if they result in patient harm | 2.87 | 1.265 | 30.7% | 2.73 | 1.31 | 28.6% | 2.91 | 1.243 | 31.1% |
| 20 | All medical errors should be reported | 4.27 | .910 | 80.2% | 4.34 | .888 | 85.2% | 4.25 | .916 | 78.7% |
| Team functioning | | 4.58 | .62 |  | 4.64 | .57 |  | 4.57 | .63 |  |
| 22 | Better multi-disciplinary teamwork will reduce medical errors | 4.56 | .71 | 91.1% | 4.58 | .738 | 91.4% | 4.56 | .705 | 91.1% |
| 23 | Teaching teamwork skills will reduce medical errors | 4.62 | .63 | 93% | 4.71 | .537 | 96% | 4.59 | .666 | 92% |
| Patient involvement in reducing errors | | 3.98 | .83 |  | 4.14 | .76 |  | 3.92 | .85 |  |
| 24 | Patients have an important role in preventing medical errors | 3.79 | 1.03 | 60.3% | 3.94 | .972 | 66.9% | 3.74 | 1.06 | 57.6% |
| 25 | Encouraging patients to be more involved in their care can help to reduce the risk of medical errors occurring | 4.18 | .835 | 80.4% | 4.35 | .757 | 85.2% | 4.12 | .856 | 78.7% |
| Importance of patient safety in the curriculum | | 4.03 | .57 |  | 4.04 | .59 |  | 4.03 | .56 |  |
| 26 | Teaching residents about patient safety should be an important priority in residency training. | 4.68 | .610 | 93.8% | 4.67 | .580 | 94.2% | 4.69 | .619 | 93.7% |
| 27 | Patient safety issues cannot be taught and can only be learned by clinical experience when qualified* | 2.99 | 1.22 | 37.6% | 3.00 | 1.25 | 36.6% | 2.98 | 1.21 | 37.5% |
| 28 | Learning about patient safety issues before I qualify will enable me to become a more effective specialist | 4.43 | .770 | 87.9% | 4.46 | .756 | 89.2% | 4.42 | .775 | 87.6% |

* Reverse items (% disagree and strongly disagree were reported) ** Higher scores indicated more affirmative responses
